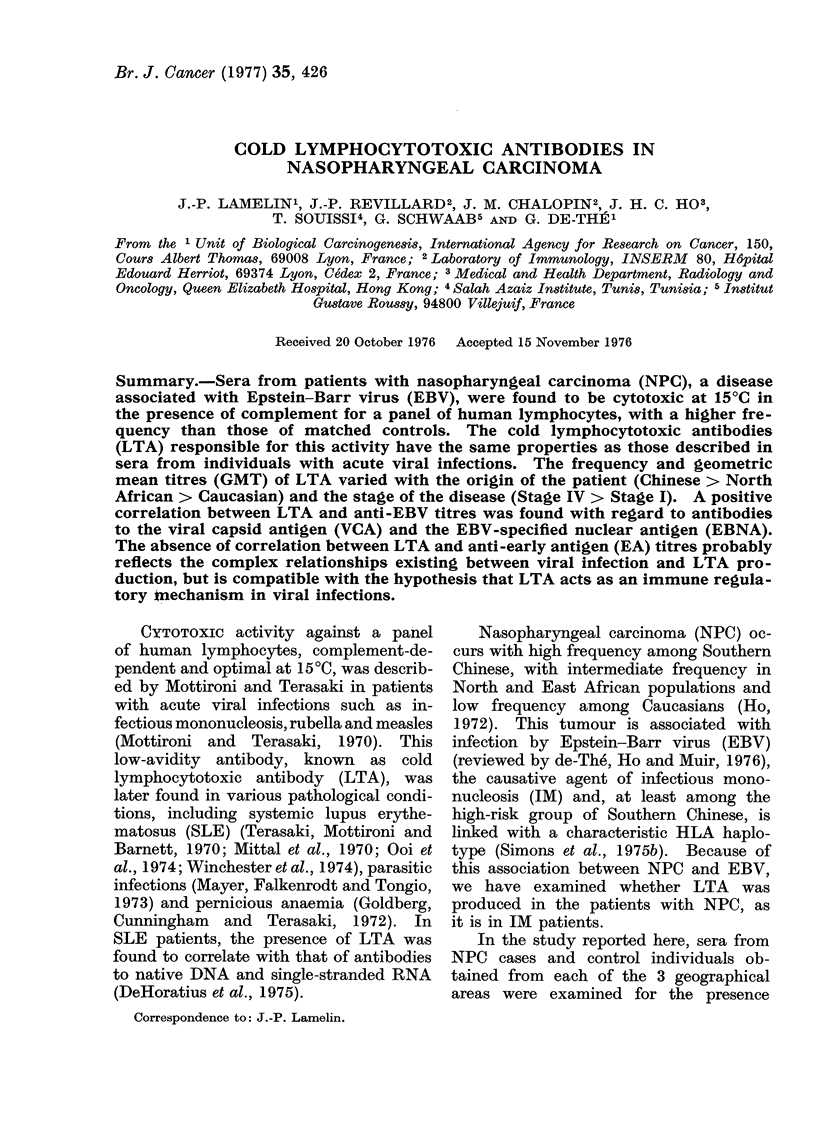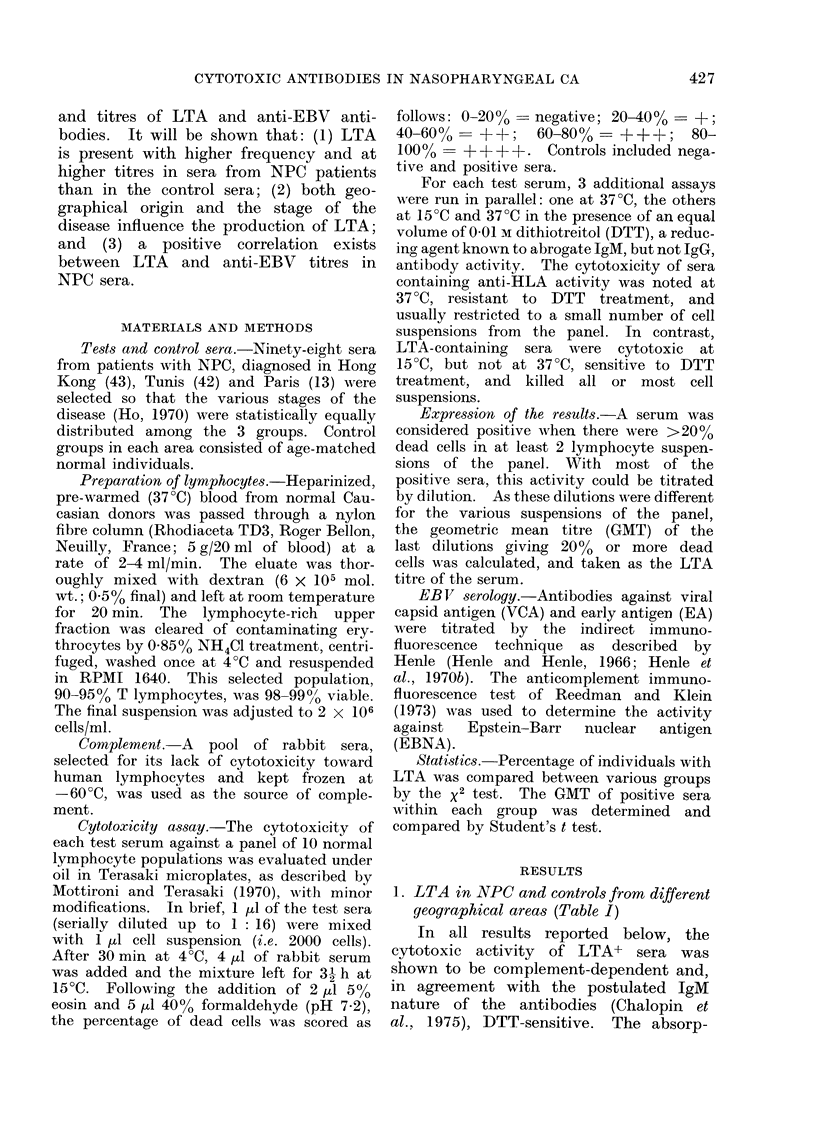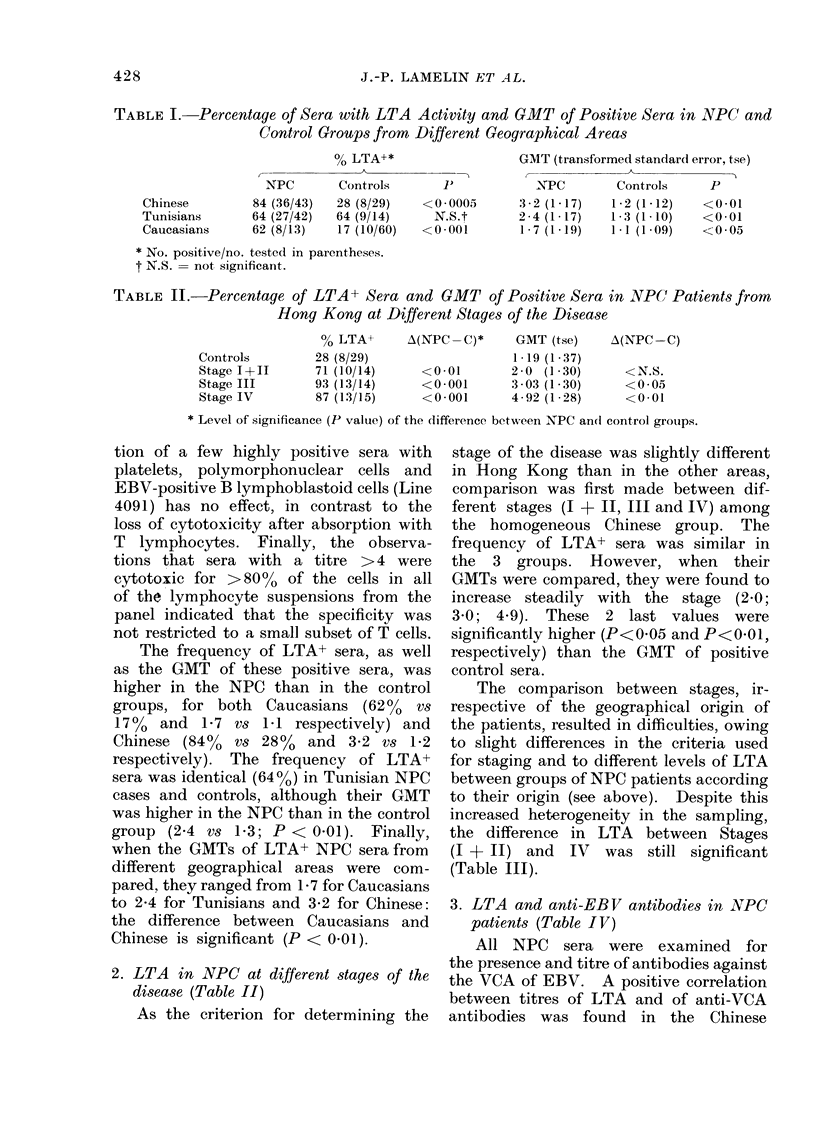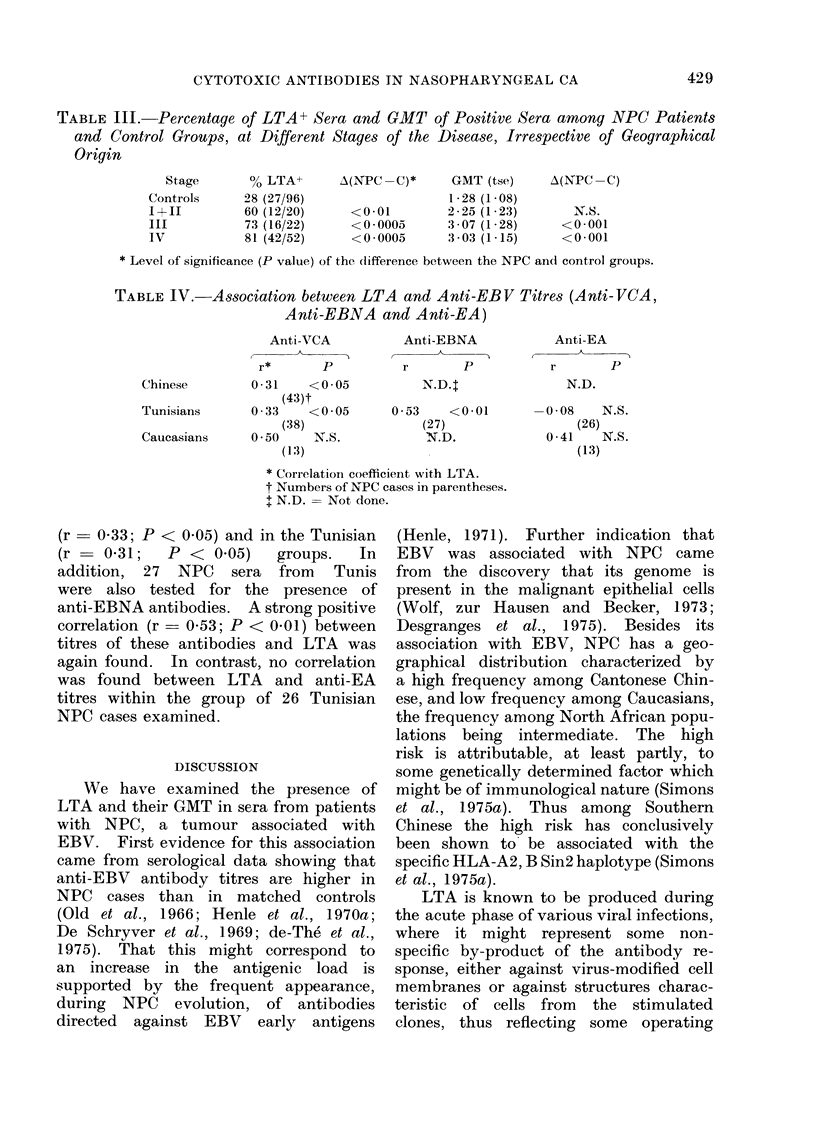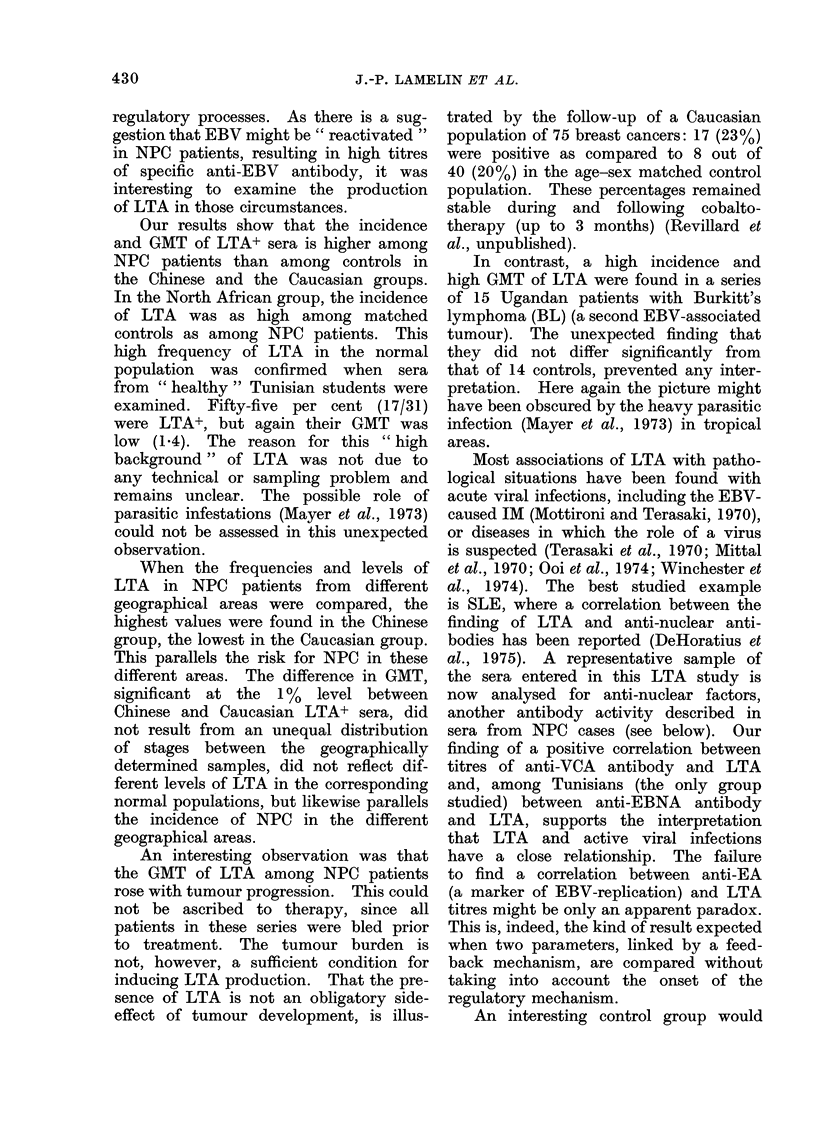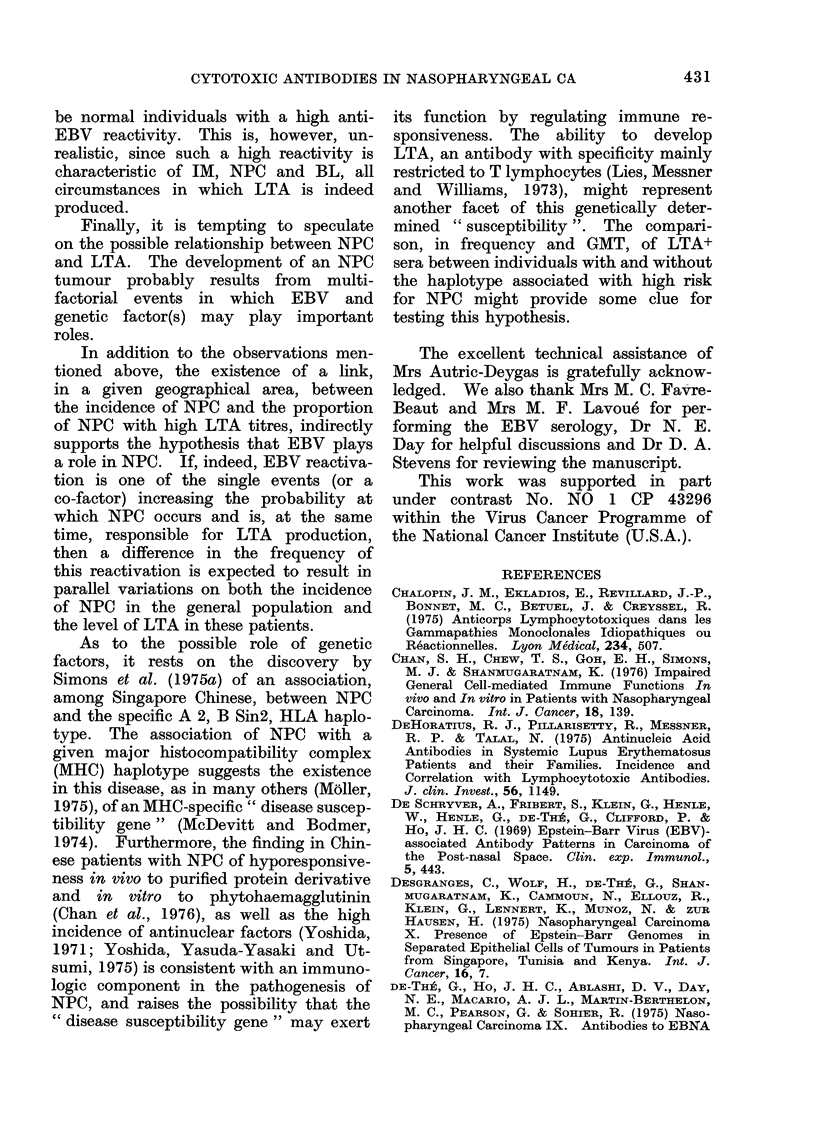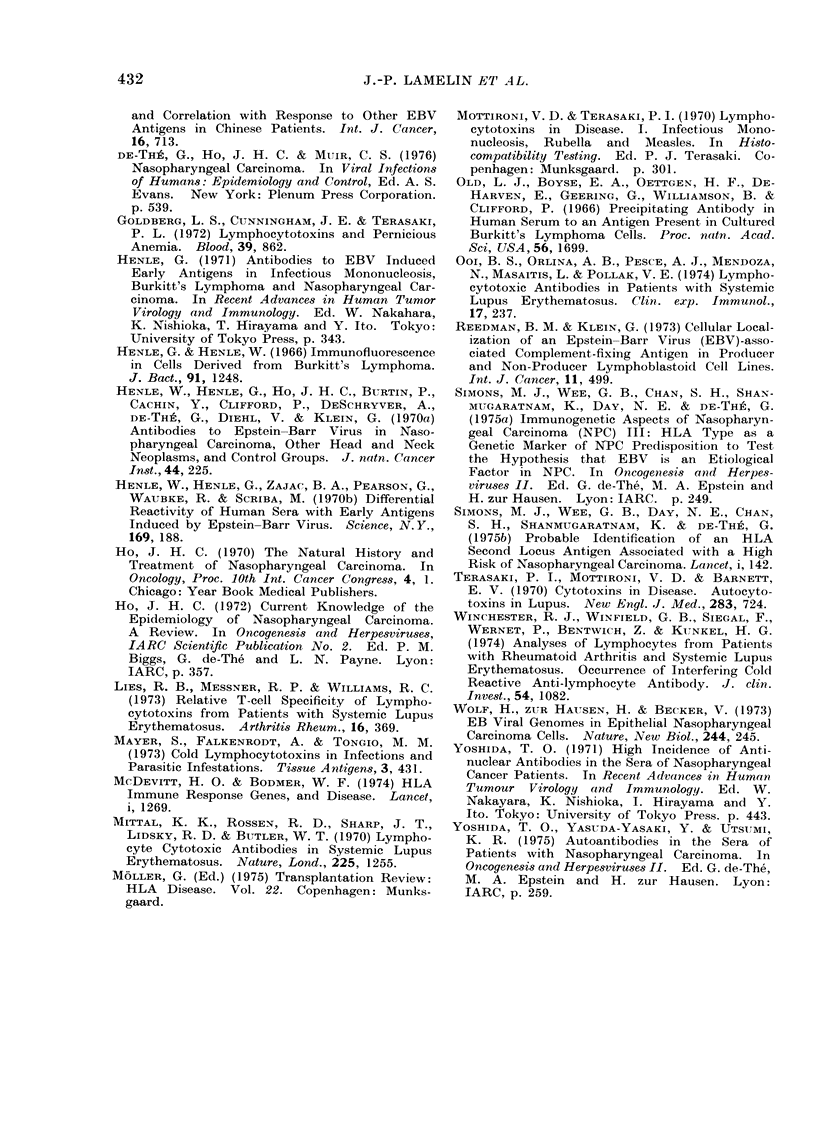# Cold lymphocytotoxic antibodies in nasopharyngeal carcinoma.

**DOI:** 10.1038/bjc.1977.64

**Published:** 1977-04

**Authors:** J. P. Lamelin, J. P. Revillard, J. M. Chalopin, J. H. Ho, T. Souissi, G. Schwaab, G. De-Thé

## Abstract

Sera from patients with nasopharyngeal carcinoma (NPC), a disease associated with Epstein-Barr virus (EBV), were found to be cytotoxic at 15% degrees C in the presence of complement for a panel of human lymphocytes, with a higher frequency than those of matched controls. The cold lymphocytotoxic antibodies (LTA) responsible for this activity have the same properties as those described in sera from individuals with acute viral infections. The frequency and geometric mean titres (GMT) of LTA varied with the origin of the patient (Chinese larger than North African larger than Caucasian) and the stage of the disease (Stage IV larger than Stage I). A positive correlation between LTA and anti-EBV titres was found with regard to antibodies to the viral capsid antigen (VCA) and the EBV-specified nuclear antigen (EBNA). The absence of correlation between LTA and anti-early antigen (EA) titres probable reflects the complex relationships existing between viral infection and LTA production, but is compatible with the hypothesis that LTA acts as an immune regulatory mechanism in viral infections.


*Br. J. Cancer* (1977) **35**, 426

### COLD LYMPHOCYTOTOXIC ANTIBODIES IN NASOPHARYNGEAL CARCINOMA

J.-P. LAMELIN[1], J.-P. REVILLARD[2], J. M. CHALOPIN[2], J. H. C. HO[3], T. SOUISSI[4], G. SCHWAAB[5] AND G. DE-THÉ[1]

*From the [1] Unit of Biological Carcinogenesis, International Agency for Research on Cancer,* 150, *Cours Albert Thomas,* 69008 *Lyon, France; [2] Laboratory of Immunology, INSERM* 80, *Hôpital Edouard Herriot,* 69374 *Lyon, Cédex* 2, *France; [3] Medical and Health Department, Radiology and Oncology, Queen Elizabeth Hospital, Hong Kong; [4] Salah Azaiz Institute, Tunis, Tunisia; [5] Institut Gustave Roussy,* 94800 *Villejuif, France*

Received 20 October 1976 Accepted 15 November 1976

**Summary.—Sera from patients with nasopharyngeal carcinoma (NPC), a disease associated with Epstein–Barr virus (EBV), were found to be cytotoxic at 15°C in the presence of complement for a panel of human lymphocytes, with a higher frequency than those of matched controls. The cold lymphocytotoxic antibodies (LTA) responsible for this activity have the same properties as those described in sera from individuals with acute viral infections. The frequency and geometric mean titres (GMT) of LTA varied with the origin of the patient (Chinese > North African > Caucasian) and the stage of the disease (Stage IV > Stage I). A positive correlation between LTA and anti-EBV titres was found with regard to antibodies to the viral capsid antigen (VCA) and the EBV-specified nuclear antigen (EBNA). The absence of correlation between LTA and anti-early antigen (EA) titres probably reflects the complex relationships existing between viral infection and LTA production, but is compatible with the hypothesis that LTA acts as an immune regulatory mechanism in viral infections.**

CYTOTOXIC activity against a panel of human lymphocytes, complement-dependent and optimal at 15°C, was described by Mottironi and Terasaki in patients with acute viral infections such as infectious mononucleosis, rubella and measles (Mottironi and Terasaki, 1970). This low-avidity antibody, known as cold lymphocytotoxic antibody (LTA), was later found in various pathological conditions, including systemic lupus erythematosus (SLE) (Terasaki, Mottironi and Barnett, 1970; Mittal *et al.*, 1970; Ooi *et al.*, 1974; Winchester *et al.*, 1974), parasitic infections (Mayer, Falkenrodt and Tongio, 1973) and pernicious anaemia (Goldberg, Cunningham and Terasaki, 1972). In SLE patients, the presence of LTA was found to correlate with that of antibodies to native DNA and single-stranded RNA (DeHoratius *et al.*, 1975).

Nasopharyngeal carcinoma (NPC) occurs with high frequency among Southern Chinese, with intermediate frequency in North and East African populations and low frequency among Caucasians (Ho, 1972). This tumour is associated with infection by Epstein–Barr virus (EBV) (reviewed by de-Thé, Ho and Muir, 1976), the causative agent of infectious mononucleosis (IM) and, at least among the high-risk group of Southern Chinese, is linked with a characteristic HLA haplotype (Simons *et al.*, 1975*b*). Because of this association between NPC and EBV, we have examined whether LTA was produced in the patients with NPC, as it is in IM patients.

In the study reported here, sera from NPC cases and control individuals obtained from each of the 3 geographical areas were examined for the presence

Correspondence to: J.-P. Lamelin.

and titres of LTA and anti-EBV antibodies. It will be shown that: (1) LTA is present with higher frequency and at higher titres in sera from NPC patients than in the control sera; (2) both geographical origin and the stage of the disease influence the production of LTA; and (3) a positive correlation exists between LTA and anti-EBV titres in NPC sera.

### MATERIALS AND METHODS

*Tests and control sera.*—Ninety-eight sera from patients with NPC, diagnosed in Hong Kong (43), Tunis (42) and Paris (13) were selected so that the various stages of the disease (Ho, 1970) were statistically equally distributed among the 3 groups. Control groups in each area consisted of age-matched normal individuals.

*Preparation of lymphocytes.*—Heparinized, pre-warmed (37 °C) blood from normal Caucasian donors was passed through a nylon fibre column (Rhodiaceta TD3, Roger Bellon, Neuilly, France; 5 g/20 ml of blood) at a rate of 2–4 ml/min. The eluate was thoroughly mixed with dextran ($6 \times 10^5$ mol. wt.; 0·5% final) and left at room temperature for 20 min. The lymphocyte-rich upper fraction was cleared of contaminating erythrocytes by 0·85% $NH_4Cl$ treatment, centrifuged, washed once at 4 °C and resuspended in RPMI 1640. This selected population, 90–95% T lymphocytes, was 98–99% viable. The final suspension was adjusted to $2 \times 10^6$ cells/ml.

*Complement.*—A pool of rabbit sera, selected for its lack of cytotoxicity toward human lymphocytes and kept frozen at −60 °C, was used as the source of complement.

*Cytotoxicity assay.*—The cytotoxicity of each test serum against a panel of 10 normal lymphocyte populations was evaluated under oil in Terasaki microplates, as described by Mottironi and Terasaki (1970), with minor modifications. In brief, 1 $\mu$l of the test sera (serially diluted up to 1 : 16) were mixed with 1 $\mu$l cell suspension (*i.e.* 2000 cells). After 30 min at 4 °C, 4 $\mu$l of rabbit serum was added and the mixture left for $3\frac{1}{2}$ h at 15 °C. Following the addition of 2 $\mu$l 5% eosin and 5 $\mu$l 40% formaldehyde (pH 7·2), the percentage of dead cells was scored as follows: 0–20% = negative; 20–40% = +; 40–60% = ++; 60–80% = +++; 80–100% = ++++. Controls included negative and positive sera.

For each test serum, 3 additional assays were run in parallel: one at 37 °C, the others at 15 °C and 37 °C in the presence of an equal volume of 0·01 M dithiotreitol (DTT), a reducing agent known to abrogate IgM, but not IgG, antibody activity. The cytotoxicity of sera containing anti-HLA activity was noted at 37 °C, resistant to DTT treatment, and usually restricted to a small number of cell suspensions from the panel. In contrast, LTA-containing sera were cytotoxic at 15 °C, but not at 37 °C, sensitive to DTT treatment, and killed all or most cell suspensions.

*Expression of the results.*—A serum was considered positive when there were >20% dead cells in at least 2 lymphocyte suspensions of the panel. With most of the positive sera, this activity could be titrated by dilution. As these dilutions were different for the various suspensions of the panel, the geometric mean titre (GMT) of the last dilutions giving 20% or more dead cells was calculated, and taken as the LTA titre of the serum.

*EBV serology.*—Antibodies against viral capsid antigen (VCA) and early antigen (EA) were titrated by the indirect immunofluorescence technique as described by Henle (Henle and Henle, 1966; Henle *et al.*, 1970*b*). The anticomplement immunofluorescence test of Reedman and Klein (1973) was used to determine the activity against Epstein–Barr nuclear antigen (EBNA).

*Statistics.*—Percentage of individuals with LTA was compared between various groups by the $\chi^2$ test. The GMT of positive sera within each group was determined and compared by Student's *t* test.

### RESULTS

### 1. *LTA in NPC and controls from different geographical areas (Table I)*

In all results reported below, the cytotoxic activity of $LTA^+$ sera was shown to be complement-dependent and, in agreement with the postulated IgM nature of the antibodies (Chalopin *et al.*, 1975), DTT-sensitive. The absorp-

TABLE I.—*Percentage of Sera with LTA Activity and GMT of Positive Sera in NPC and Control Groups from Different Geographical Areas*

| | % LTA+* | | | GMT (transformed standard error, tse) | | |
|---|---|---|---|---|---|---|
| | NPC | Controls | *P* | NPC | Controls | *P* |
| Chinese | 84 (36/43) | 28 (8/29) | <0·0005 | 3·2 (1·17) | 1·2 (1·12) | <0·01 |
| Tunisians | 64 (27/42) | 64 (9/14) | N.S.† | 2·4 (1·17) | 1·3 (1·10) | <0·01 |
| Caucasians | 62 (8/13) | 17 (10/60) | <0·001 | 1·7 (1·19) | 1·1 (1·09) | <0·05 |

* No. positive/no. tested in parentheses.
† N.S. = not significant.

TABLE II.—*Percentage of LTA+ Sera and GMT of Positive Sera in NPC Patients from Hong Kong at Different Stages of the Disease*

| | % LTA+ | Δ(NPC−C)* | GMT (tse) | Δ(NPC−C) |
|---|---|---|---|---|
| Controls | 28 (8/29) | | 1·19 (1·37) | |
| Stage I+II | 71 (10/14) | <0·01 | 2·0 (1·30) | <N.S. |
| Stage III | 93 (13/14) | <0·001 | 3·03 (1·30) | <0·05 |
| Stage IV | 87 (13/15) | <0·001 | 4·92 (1·28) | <0·01 |

* Level of significance (*P* value) of the difference between NPC and control groups.

tion of a few highly positive sera with platelets, polymorphonuclear cells and EBV-positive B lymphoblastoid cells (Line 4091) has no effect, in contrast to the loss of cytotoxicity after absorption with T lymphocytes. Finally, the observations that sera with a titre >4 were cytotoxic for >80% of the cells in all of the lymphocyte suspensions from the panel indicated that the specificity was not restricted to a small subset of T cells.

The frequency of LTA+ sera, as well as the GMT of these positive sera, was higher in the NPC than in the control groups, for both Caucasians (62% *vs* 17% and 1·7 *vs* 1·1 respectively) and Chinese (84% *vs* 28% and 3·2 *vs* 1·2 respectively). The frequency of LTA+ sera was identical (64%) in Tunisian NPC cases and controls, although their GMT was higher in the NPC than in the control group (2·4 *vs* 1·3; $P < 0·01$). Finally, when the GMTs of LTA+ NPC sera from different geographical areas were compared, they ranged from 1·7 for Caucasians to 2·4 for Tunisians and 3·2 for Chinese: the difference between Caucasians and Chinese is significant ($P < 0·01$).

2. *LTA in NPC at different stages of the disease* (*Table II*)

As the criterion for determining the stage of the disease was slightly different in Hong Kong than in the other areas, comparison was first made between different stages (I + II, III and IV) among the homogeneous Chinese group. The frequency of LTA+ sera was similar in the 3 groups. However, when their GMTs were compared, they were found to increase steadily with the stage (2·0; 3·0; 4·9). These 2 last values were significantly higher ($P<0·05$ and $P<0·01$, respectively) than the GMT of positive control sera.

The comparison between stages, irrespective of the geographical origin of the patients, resulted in difficulties, owing to slight differences in the criteria used for staging and to different levels of LTA between groups of NPC patients according to their origin (see above). Despite this increased heterogeneity in the sampling, the difference in LTA between Stages (I + II) and IV was still significant (Table III).

3. *LTA and anti-EBV antibodies in NPC patients* (*Table IV*)

All NPC sera were examined for the presence and titre of antibodies against the VCA of EBV. A positive correlation between titres of LTA and of anti-VCA antibodies was found in the Chinese

TABLE III.—*Percentage of $LTA^+$ Sera and GMT of Positive Sera among NPC Patients and Control Groups, at Different Stages of the Disease, Irrespective of Geographical Origin*

| Stage | % $LTA^+$ | Δ(NPC−C)* | GMT (tse) | Δ(NPC−C) |
|---|---|---|---|---|
| Controls | 28 (27/96) | | 1·28 (1·08) | |
| I+II | 60 (12/20) | <0·01 | 2·25 (1·23) | N.S. |
| III | 73 (16/22) | <0·0005 | 3·07 (1·28) | <0·001 |
| IV | 81 (42/52) | <0·0005 | 3·03 (1·15) | <0·001 |

* Level of significance (*P* value) of the difference between the NPC and control groups.

TABLE IV.—*Association between LTA and Anti-EBV Titres (Anti-VCA, Anti-EBNA and Anti-EA)*

| | Anti-VCA | | Anti-EBNA | | Anti-EA | |
|---|---|---|---|---|---|---|
| | r* | *P* | r | *P* | r | *P* |
| Chinese | 0·31 (43)† | <0·05 | N.D.‡ | | N.D. | |
| Tunisians | 0·33 (38) | <0·05 | 0·53 (27) | <0·01 | −0·08 (26) | N.S. |
| Caucasians | 0·50 (13) | N.S. | N.D. | | 0·41 (13) | N.S. |

* Correlation coefficient with LTA.
† Numbers of NPC cases in parentheses.
‡ N.D. = Not done.

(r = 0·33; $P < 0·05$) and in the Tunisian (r = 0·31; $P < 0·05$) groups. In addition, 27 NPC sera from Tunis were also tested for the presence of anti-EBNA antibodies. A strong positive correlation (r = 0·53; $P < 0·01$) between titres of these antibodies and LTA was again found. In contrast, no correlation was found between LTA and anti-EA titres within the group of 26 Tunisian NPC cases examined.

### DISCUSSION

We have examined the presence of LTA and their GMT in sera from patients with NPC, a tumour associated with EBV. First evidence for this association came from serological data showing that anti-EBV antibody titres are higher in NPC cases than in matched controls (Old *et al.*, 1966; Henle *et al.*, 1970*a*; De Schryver *et al.*, 1969; de-Thé *et al.*, 1975). That this might correspond to an increase in the antigenic load is supported by the frequent appearance, during NPC evolution, of antibodies directed against EBV early antigens (Henle, 1971). Further indication that EBV was associated with NPC came from the discovery that its genome is present in the malignant epithelial cells (Wolf, zur Hausen and Becker, 1973; Desgranges *et al.*, 1975). Besides its association with EBV, NPC has a geographical distribution characterized by a high frequency among Cantonese Chinese, and low frequency among Caucasians, the frequency among North African populations being intermediate. The high risk is attributable, at least partly, to some genetically determined factor which might be of immunological nature (Simons *et al.*, 1975*a*). Thus among Southern Chinese the high risk has conclusively been shown to be associated with the specific HLA-A2, B Sin2 haplotype (Simons *et al.*, 1975*a*).

LTA is known to be produced during the acute phase of various viral infections, where it might represent some non-specific by-product of the antibody response, either against virus-modified cell membranes or against structures characteristic of cells from the stimulated clones, thus reflecting some operating regulatory processes. As there is a suggestion that EBV might be " reactivated " in NPC patients, resulting in high titres of specific anti-EBV antibody, it was interesting to examine the production of LTA in those circumstances.

Our results show that the incidence and GMT of $LTA^+$ sera is higher among NPC patients than among controls in the Chinese and the Caucasian groups. In the North African group, the incidence of LTA was as high among matched controls as among NPC patients. This high frequency of LTA in the normal population was confirmed when sera from " healthy " Tunisian students were examined. Fifty-five per cent (17/31) were $LTA^+$, but again their GMT was low (1·4). The reason for this " high background " of LTA was not due to any technical or sampling problem and remains unclear. The possible role of parasitic infestations (Mayer *et al.*, 1973) could not be assessed in this unexpected observation.

When the frequencies and levels of LTA in NPC patients from different geographical areas were compared, the highest values were found in the Chinese group, the lowest in the Caucasian group. This parallels the risk for NPC in these different areas. The difference in GMT, significant at the 1% level between Chinese and Caucasian $LTA^+$ sera, did not result from an unequal distribution of stages between the geographically determined samples, did not reflect different levels of LTA in the corresponding normal populations, but likewise parallels the incidence of NPC in the different geographical areas.

An interesting observation was that the GMT of LTA among NPC patients rose with tumour progression. This could not be ascribed to therapy, since all patients in these series were bled prior to treatment. The tumour burden is not, however, a sufficient condition for inducing LTA production. That the presence of LTA is not an obligatory side-effect of tumour development, is illustrated by the follow-up of a Caucasian population of 75 breast cancers: 17 (23%) were positive as compared to 8 out of 40 (20%) in the age–sex matched control population. These percentages remained stable during and following cobaltotherapy (up to 3 months) (Revillard *et al.*, unpublished).

In contrast, a high incidence and high GMT of LTA were found in a series of 15 Ugandan patients with Burkitt's lymphoma (BL) (a second EBV-associated tumour). The unexpected finding that they did not differ significantly from that of 14 controls, prevented any interpretation. Here again the picture might have been obscured by the heavy parasitic infection (Mayer *et al.*, 1973) in tropical areas.

Most associations of LTA with pathological situations have been found with acute viral infections, including the EBV-caused IM (Mottironi and Terasaki, 1970), or diseases in which the role of a virus is suspected (Terasaki *et al.*, 1970; Mittal *et al.*, 1970; Ooi *et al.*, 1974; Winchester *et al.*, 1974). The best studied example is SLE, where a correlation between the finding of LTA and anti-nuclear antibodies has been reported (DeHoratius *et al.*, 1975). A representative sample of the sera entered in this LTA study is now analysed for anti-nuclear factors, another antibody activity described in sera from NPC cases (see below). Our finding of a positive correlation between titres of anti-VCA antibody and LTA and, among Tunisians (the only group studied) between anti-EBNA antibody and LTA, supports the interpretation that LTA and active viral infections have a close relationship. The failure to find a correlation between anti-EA (a marker of EBV-replication) and LTA titres might be only an apparent paradox. This is, indeed, the kind of result expected when two parameters, linked by a feedback mechanism, are compared without taking into account the onset of the regulatory mechanism.

An interesting control group would be normal individuals with a high anti-EBV reactivity. This is, however, unrealistic, since such a high reactivity is characteristic of IM, NPC and BL, all circumstances in which LTA is indeed produced.

Finally, it is tempting to speculate on the possible relationship between NPC and LTA. The development of an NPC tumour probably results from multifactorial events in which EBV and genetic factor(s) may play important roles.

In addition to the observations mentioned above, the existence of a link, in a given geographical area, between the incidence of NPC and the proportion of NPC with high LTA titres, indirectly supports the hypothesis that EBV plays a role in NPC. If, indeed, EBV reactivation is one of the single events (or a co-factor) increasing the probability at which NPC occurs and is, at the same time, responsible for LTA production, then a difference in the frequency of this reactivation is expected to result in parallel variations on both the incidence of NPC in the general population and the level of LTA in these patients.

As to the possible role of genetic factors, it rests on the discovery by Simons *et al.* (1975*a*) of an association, among Singapore Chinese, between NPC and the specific A 2, B Sin2, HLA haplotype. The association of NPC with a given major histocompatibility complex (MHC) haplotype suggests the existence in this disease, as in many others (Möller, 1975), of an MHC-specific " disease susceptibility gene " (McDevitt and Bodmer, 1974). Furthermore, the finding in Chinese patients with NPC of hyporesponsiveness *in vivo* to purified protein derivative and *in vitro* to phytohaemagglutinin (Chan *et al.*, 1976), as well as the high incidence of antinuclear factors (Yoshida, 1971; Yoshida, Yasuda-Yasaki and Utsumi, 1975) is consistent with an immunologic component in the pathogenesis of NPC, and raises the possibility that the " disease susceptibility gene " may exert its function by regulating immune responsiveness. The ability to develop LTA, an antibody with specificity mainly restricted to T lymphocytes (Lies, Messner and Williams, 1973), might represent another facet of this genetically determined " susceptibility ". The comparison, in frequency and GMT, of LTA+ sera between individuals with and without the haplotype associated with high risk for NPC might provide some clue for testing this hypothesis.

The excellent technical assistance of Mrs Autric-Deygas is gratefully acknowledged. We also thank Mrs M. C. Favre-Beaut and Mrs M. F. Lavoué for performing the EBV serology, Dr N. E. Day for helpful discussions and Dr D. A. Stevens for reviewing the manuscript.

This work was supported in part under contrast No. NO 1 CP 43296 within the Virus Cancer Programme of the National Cancer Institute (U.S.A.).

### REFERENCES

CHALOPIN, J. M., EKLADIOS, E., REVILLARD, J.-P., BONNET, M. C., BETUEL, J. & CREYSSEL, R. (1975) Anticorps Lymphocytotoxiques dans les Gammapathies Monoclonales Idiopathiques ou Réactionnelles. *Lyon Médical*, **234**, 507.

CHAN, S. H., CHEW, T. S., GOH, E. H., SIMONS, M. J. & SHANMUGARATNAM, K. (1976) Impaired General Cell-mediated Immune Functions *In vivo* and *In vitro* in Patients with Nasopharyngeal Carcinoma. *Int. J. Cancer*, **18**, 139.

DEHORATIUS, R. J., PILLARISETTY, R., MESSNER, R. P. & TALAL, N. (1975) Antinucleic Acid Antibodies in Systemic Lupus Erythematosus Patients and their Families. Incidence and Correlation with Lymphocytotoxic Antibodies. *J. clin. Invest.*, **56**, 1149.

DE SCHRYVER, A., FRIBERT, S., KLEIN, G., HENLE, W., HENLE, G., DE-THÉ, G., CLIFFORD, P. & HO, J. H. C. (1969) Epstein–Barr Virus (EBV)-associated Antibody Patterns in Carcinoma of the Post-nasal Space. *Clin. exp. Immunol.*, **5**, 443.

DESGRANGES, C., WOLF, H., DE-THÉ, G., SHANMUGARATNAM, K., CAMMOUN, N., ELLOUZ, R., KLEIN, G., LENNERT, K., MUNOZ, N. & ZUR HAUSEN, H. (1975) Nasopharyngeal Carcinoma X. Presence of Epstein–Barr Genomes in Separated Epithelial Cells of Tumours in Patients from Singapore, Tunisia and Kenya. *Int. J. Cancer*, **16**, 7.

DE-THÉ, G., HO, J. H. C., ABLASHI, D. V., DAY, N. E., MACARIO, A. J. L., MARTIN-BERTHELON, M. C., PEARSON, G. & SOHIER, R. (1975) Nasopharyngeal Carcinoma IX. Antibodies to EBNA

and Correlation with Response to Other EBV Antigens in Chinese Patients. *Int. J. Cancer*, **16**, 713.

DE-THÉ, G., HO, J. H. C. & MUIR, C. S. (1976) Nasopharyngeal Carcinoma. In *Viral Infections of Humans: Epidemiology and Control*, Ed. A. S. Evans. New York: Plenum Press Corporation. p. 539.

GOLDBERG, L. S., CUNNINGHAM, J. E. & TERASAKI, P. L. (1972) Lymphocytotoxins and Pernicious Anemia. *Blood*, **39**, 862.

HENLE, G. (1971) Antibodies to EBV Induced Early Antigens in Infectious Mononucleosis, Burkitt's Lymphoma and Nasopharyngeal Carcinoma. In *Recent Advances in Human Tumor Virology and Immunology*. Ed. W. Nakahara, K. Nishioka, T. Hirayama and Y. Ito. Tokyo: University of Tokyo Press, p. 343.

HENLE, G. & HENLE, W. (1966) Immunofluorescence in Cells Derived from Burkitt's Lymphoma. *J. Bact.*, **91**, 1248.

HENLE, W., HENLE, G., HO, J. H. C., BURTIN, P., CACHIN, Y., CLIFFORD, P., DESCHRYVER, A., DE-THÉ, G., DIEHL, V. & KLEIN, G. (1970*a*) Antibodies to Epstein–Barr Virus in Nasopharyngeal Carcinoma, Other Head and Neck Neoplasms, and Control Groups. *J. natn. Cancer Inst.*, **44**, 225.

HENLE, W., HENLE, G., ZAJAC, B. A., PEARSON, G., WAUBKE, R. & SCRIBA, M. (1970b) Differential Reactivity of Human Sera with Early Antigens Induced by Epstein–Barr Virus. *Science, N.Y.*, **169**, 188.

HO, J. H. C. (1970) The Natural History and Treatment of Nasopharyngeal Carcinoma. In *Oncology, Proc. 10th Int. Cancer Congress*, **4**, 1. Chicago: Year Book Medical Publishers.

HO, J. H. C. (1972) Current Knowledge of the Epidemiology of Nasopharyngeal Carcinoma. A Review. In *Oncogenesis and Herpesviruses, IARC Scientific Publication No. 2*. Ed. P. M. Biggs, G. de-Thé and L. N. Payne. Lyon: IARC, p. 357.

LIES, R. B., MESSNER, R. P. & WILLIAMS, R. C. (1973) Relative T-cell Specificity of Lymphocytotoxins from Patients with Systemic Lupus Erythematosus. *Arthritis Rheum.*, **16**, 369.

MAYER, S., FALKENRODT, A. & TONGIO, M. M. (1973) Cold Lymphocytotoxins in Infections and Parasitic Infestations. *Tissue Antigens*, **3**, 431.

MCDEVITT, H. O. & BODMER, W. F. (1974) HLA Immune Response Genes, and Disease. *Lancet*, i, 1269.

MITTAL, K. K., ROSSEN, R. D., SHARP, J. T., LIDSKY, R. D. & BUTLER, W. T. (1970) Lymphocyte Cytotoxic Antibodies in Systemic Lupus Erythematosus. *Nature, Lond.*, **225**, 1255.

MÖLLER, G. (Ed.) (1975) Transplantation Review: HLA Disease. Vol. *22*. Copenhagen: Munksgaard.

MOTTIRONI, V. D. & TERASAKI, P. I. (1970) Lymphocytotoxins in Disease. I. Infectious Mononucleosis, Rubella and Measles. In *Histocompatibility Testing*. Ed. P. J. Terasaki. Copenhagen: Munksgaard. p. 301.

OLD, L. J., BOYSE, E. A., OETTGEN, H. F., DE-HARVEN, E., GEERING, G., WILLIAMSON, B. & CLIFFORD, P. (1966) Precipitating Antibody in Human Serum to an Antigen Present in Cultured Burkitt's Lymphoma Cells. *Proc. natn. Acad. Sci, USA*, **56**, 1699.

OOI, B. S., ORLINA, A. B., PESCE, A. J., MENDOZA, N., MASAITIS, L. & POLLAK, V. E. (1974) Lymphocytotoxic Antibodies in Patients with Systemic Lupus Erythematosus. *Clin. exp. Immunol.*, **17**, 237.

REEDMAN, B. M. & KLEIN, G. (1973) Cellular Localization of an Epstein–Barr Virus (EBV)-associated Complement-fixing Antigen in Producer and Non-Producer Lymphoblastoid Cell Lines. *Int. J. Cancer*, **11**, 499.

SIMONS, M. J., WEE, G. B., CHAN, S. H., SHANMUGARATNAM, K., DAY, N. E. & DE-THÉ, G. (1975*a*) Immunogenetic Aspects of Nasopharyngeal Carcinoma (NPC) III: HLA Type as a Genetic Marker of NPC Predisposition to Test the Hypothesis that EBV is an Etiological Factor in NPC. In *Oncogenesis and Herpesviruses II*. Ed. G. de-Thé, M. A. Epstein and H. zur Hausen. Lyon: IARC. p. 249.

SIMONS, M. J., WEE, G. B., DAY, N. E., CHAN, S. H., SHANMUGARATNAM, K. & DE-THÉ, G. (1975*b*) Probable Identification of an HLA Second Locus Antigen Associated with a High Risk of Nasopharyngeal Carcinoma. *Lancet*, i, 142.

TERASAKI, P. I., MOTTIRONI, V. D. & BARNETT, E. V. (1970) Cytotoxins in Disease. Autocytotoxins in Lupus. *New Engl. J. Med.*, **283**, 724.

WINCHESTER, R. J., WINFIELD, G. B., SIEGAL, F., WERNET, P., BENTWICH, Z. & KUNKEL, H. G. (1974) Analyses of Lymphocytes from Patients with Rheumatoid Arthritis and Systemic Lupus Erythematosus. Occurrence of Interfering Cold Reactive Anti-lymphocyte Antibody. *J. clin. Invest.*, **54**, 1082.

WOLF, H., ZUR HAUSEN, H. & BECKER, V. (1973) EB Viral Genomes in Epithelial Nasopharyngeal Carcinoma Cells. *Nature, New Biol.*, **244**, 245.

YOSHIDA, T. O. (1971) High Incidence of Antinuclear Antibodies in the Sera of Nasopharyngeal Cancer Patients. In *Recent Advances in Human Tumour Virology and Immunology*. Ed. W. Nakayara, K. Nishioka, I. Hirayama and Y. Ito. Tokyo: University of Tokyo Press. p. 443.

YOSHIDA, T. O., YASUDA-YASAKI, Y. & UTSUMI, K. R. (1975) Autoantibodies in the Sera of Patients with Nasopharyngeal Carcinoma. In *Oncogenesis and Herpesviruses II*. Ed. G. de-Thé, M. A. Epstein and H. zur Hausen. Lyon: IARC, p. 259.